# Alteration of coastal productivity and artisanal fisheries interact to affect a marine food web

**DOI:** 10.1038/s41598-021-81392-4

**Published:** 2021-01-19

**Authors:** M. Isidora Ávila-Thieme, Derek Corcoran, Alejandro Pérez-Matus, Evie A. Wieters, Sergio A. Navarrete, Pablo A. Marquet, Fernanda S. Valdovinos

**Affiliations:** 1 Departamento de Ecología, Facultad de Ciencias Biológicas, Pontificia Universidad Católica de Chile, Alameda 340, 8331150 Santiago, Chile; 2 Instituto de Ecología y Biodiversidad (IEB), Las Palmera 345, Santiago, Chile; 3 Universidad Católica de la Santísima Concepción, Concepción, Chile; 4 Subtidal Ecology Laboratory, Estación Costera de Investigaciones Marinas, Departamento de Ecología, Facultad de Ciencias Biológicas Pontificia, Universidad Católica de Chile, Santiago, Casilla 114-D, Santiago, Chile; 5 Estación Costera de Investigaciones Marinas - Las Cruces, Facultad de Ciencias Biológicas, Pontificia Universidad Católica de Chile, Las cruces, Chile; 6 Centro de Ecología Aplicada y Sustentabilidad (CAPES), Facultad de Ciencias Biológicas, Pontificia Universidad Católica de Chile, Alameda 340, 8331150 Santiago, Chile; 7 Centro de Cambio Global UC (PUCGlobal), Alameda 340, 8331150 Santiago, Chile; 8 The Santa Fe Institute, 1399 Hyde Park Road, Santa Fe, NM 87501 USA; 9 Instituto de Sistemas Complejos de Valparaíso (ISCV), Artillería 470, Cerro Artillería, Valparaíso, Chile; 10 Department of Environmental Science and Policy, University of California, Davis, USA

**Keywords:** Ecology, Ecological modelling, Ecological networks

## Abstract

Top-down and bottom-up forces determine ecosystem function and dynamics. Fisheries as a top-down force can shorten and destabilize food webs, while effects driven by climate change can alter the bottom-up forces of primary productivity. We assessed the response of a highly-resolved intertidal food web to these two global change drivers, using network analysis and bioenergetic modelling. We quantified the relative importance of artisanal fisheries as another predator species, and evaluated the independent and combined effects of fisheries and changes in plankton productivity on food web dynamics. The food web was robust to the loss of all harvested species but sensitive to the decline in plankton productivity. Interestingly, fisheries dampened the negative impacts of decreasing plankton productivity on non-harvested species by reducing the predation pressure of harvested consumers on non-harvested resources, and reducing the interspecific competition between harvested and non-harvested basal species. In contrast, the decline in plankton productivity increased the sensitivity of harvested species to fishing by reducing the total productivity of the food web. Our results show that strategies for new scenarios caused by climate change are needed to protect marine ecosystems and the wellbeing of local communities dependent on their resources.

## Introduction

Direct human impacts and the full suite of drivers of global change are the main cause of species extinctions in Anthropocene ecosystems^1,2^, with detrimental consequences on ecosystem functioning and their services to human societies^3,4^. The world fisheries crisis is among those consequences, which cuts across fishing strategies, oceanic regions, species, and includes countries that have little regulation and those that have implemented rights-based co-management strategies to reduce overharvesting^5–8^. Chile has been one of the countries implementing Territorial Use Rights (TURFs^9^) over an unprecedented geographic scale to manage the diverse coastal benthic resources using a co-management strategy^10,11^. These TURFS are used for artisanal fisheries, that is, fisheries that use simple fishing gears and small vessels. Over 60 coastal benthic species are actively harvested by these artisanal fisheries^10^, with species that are extracted from intertidal and shallow subtidal habitats^12,13^. The Chilean TURFs system brought significant improvements in sustainability of this complex socio-ecological system, helping to rebuild benthic fish stocks^10,11^, improving fishers’ perception towards sustainability and increasing compliance^9^, as well as showing positive ancillary effects on conservation of biodiversity^14,15^. However, the situation of most artisanal fisheries is still far from sustainable, and many fish stocks and coastal ecosystems show signs of over exploitation and ecosystem degradation, a consequence of the low levels of cooperation and low enforcement of TURF regulations, which leads to high levels of free-riding and illegal fishing^16–18^. Thus, it is imperative to improve our understanding of the effects of these multi-species fisheries which simultaneously harvest species at all trophic levels, from kelp primary producers to top carnivores^13,19^.

To compound things, removal of biomass from the ocean occurs simultaneously with multiple other stressors associated to climate change that compromise the capacity of these socio-ecological systems to respond to perturbations^20–22^. Besides sea surface temperature, climate change also affects many other physical–chemical characteristics of marine coastal waters (stratification, acidification, ventilation)^23,24^, as well as the wind regimes that control surface water productivity along the productive coastal upwelling ecosystems^25–29^. Changes in the productivity of the oceans are reflected in changes of plankton biomass. Plankton contributes approximately half of the global primary production, supports marine food webs, influences the biogeochemical process in the ocean, and strongly affects commercial fisheries^30–32^. Indeed, an overall decrease in marine plankton productivity is expected over global scales^24,30,33^. Long-term increases and decreases in plankton productivity have already occurred over the past two decades^34,35^ along extensive regions of the Humboldt upwelling ecosystem off Chile, and are expected to propagate up the pelagic and benthic food webs. We therefore analyze the bottom-up impact of fluctuations in plankton productivity in combination with fisheries exploitation of these food webs, using the concepts and methods of network ecology.

Network ecology has advanced our understanding of ecosystems by providing a powerful framework to analyze biological communities^36,37^. Previous studies used this framework to assess food web robustness against species extinctions, defined as the fraction of initial species that remain present in the ecosystem after a primary extinction^38–45^. These studies showed the importance for food web persistence of highly connected species (independent of trophic position)^38,40,46,47^, basal species^39^, and highly connected species that, at the same time, trophically support other highly connected species^42^. Most of these studies used a static approach, which stems from network theory and analyzes the impacts of structural changes on food webs represented by nodes (species) and links (interactions) that connect nodes, but ignores interaction strengths and population dynamics of interacting species^38^. Other studies used a dynamic approach, which considers not only the structure and intensity of interactions in a food web, but also the changes in species biomasses through time and the indirect effects that these changes have on other species^39–41,48–50^. Here, we use both approaches to understand the relative importance of harvested species in our food web.

In this contribution, we analyze (1) the importance of harvested species for the structure and persistence of the intertidal food web by simulating a scenario of all harvested species going extinct due to their over-exploitation by fisheries. We then evaluate (2) the robustness of this food web to the extinction of species harvested by artisanal fisheries in comparison to three commonly used extinction sequences (see below), and (3) the effects of three fisheries scenarios on other species abundance, persistence and food web dynamics. We finally analyze the (4) independent and (5) combined effects of fisheries and changes in plankton productivity on the food web dynamics through altering the plankton subsidy.

## Results

### Food web description and the relative importance of harvested species to the food web structure

**Figure 1:**
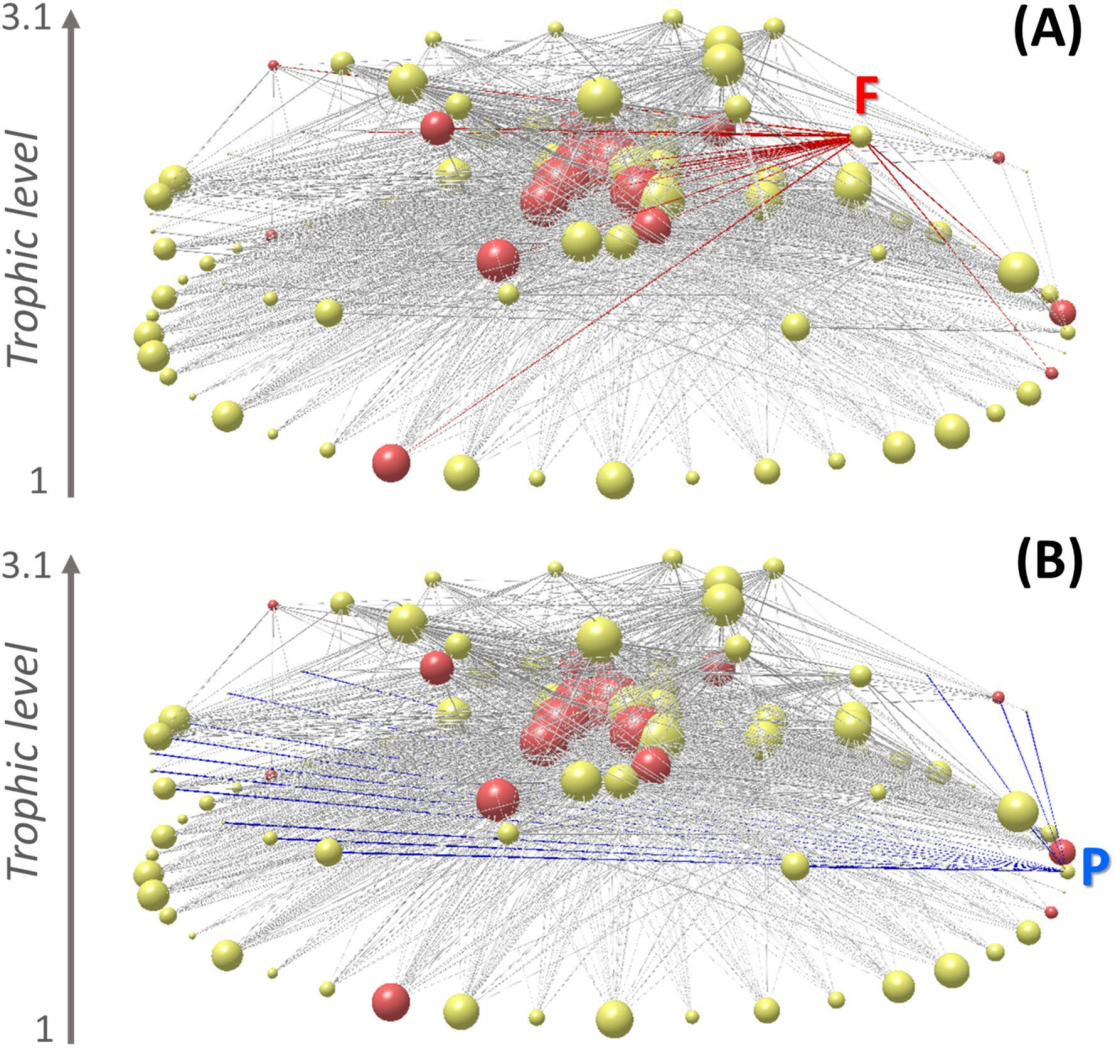
Intertidal food web highlighting nodes and links of artisanal fisheries (**A**) and plankton (**B**). Red and yellow nodes represent harvested and non-harvested species, respectively. Fisheries’ links are highlighted in red, while plankton’s links in blue. Letter F and P represent the fisheries and plankton node, respectively. Node size represents the number of trophic interactions (degree) of each node. Nodes at the bottom of the food web represent basal species, while nodes at the top of the food web represent top predators. Y-axis represents trophic level, obtained from the minimum to the maximum value of the SWTL used to calculate the MeanSWTL (see “Methods”). Drawn using Network3D software^51^.

The intertidal food web contains 107 species and 1381 trophic links, with the highly omnivorous fisheries node (F node in Fig. 1A) contributing 22 links, from basal kelp species to top carnivores (Fig. 1). Among the species harvested by the artisanal fisheries, 10 belong to the 30 most connected species of the food web (Fig. 1A; Supplementary Table S1). Moreover, these fisheries exploit at least one species that is a resource or a consumer of about 70% of the species (harvested and non-harvested species) in the intertidal food web (Supplementary Fig. S1A–C). With the static approach, we found that the removal of all 22 harvested species (see “Methods”) negatively affected the structural properties of the food web, specifically, reducing the overall number of trophic interactions by 48%. This loss represents, on average, 100 more links lost than that expected from randomly removing 22 species from the food web (see Supplementary Table S2 and Supplementary material for more detailed results).

### Food web robustness to species extinctions

**Figure 2:**
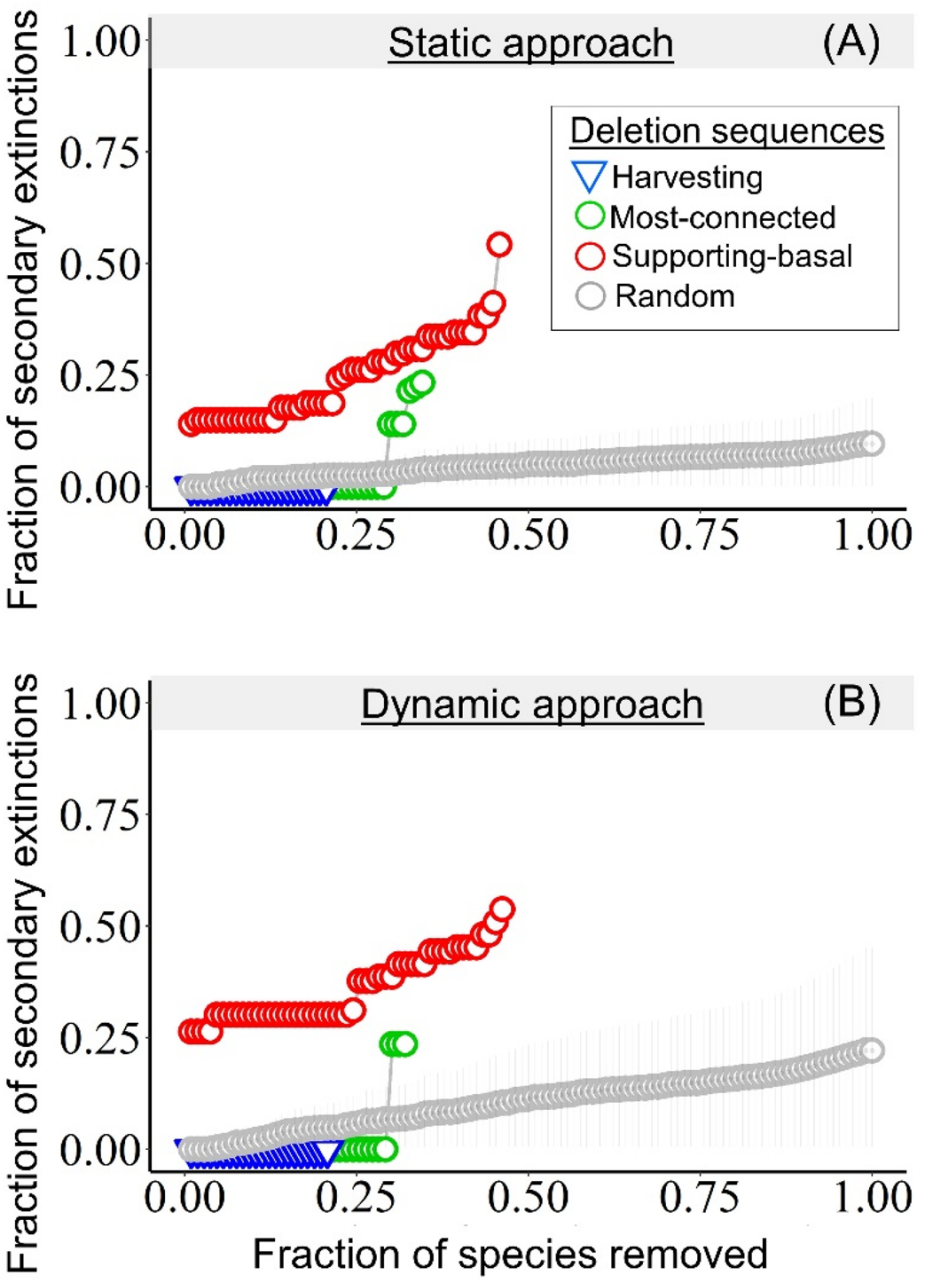
Fraction of secondary extinctions (y-axis) produced by the sequential removal of species (x-axis) with static (**A**) and dynamic (**B**) approaches. Green and red circles represent most-connected and supporting-basal deletion sequences, while blue triangle represents harvesting deletion sequence. In the random deletion sequence, circles represent the average and the error bars represent the 95% confidence interval over 1000 simulations.

Following previous work^38–41^, we evaluated the robustness of the intertidal food web to species extinction by sequentially removing species and counting the subsequent secondary extinctions, if any. We counted the secondary extinctions caused by the four deletion sequences (harvesting, random, most-connected, supporting-basal) using both static and dynamic approaches (see “Methods”). Our dynamic approach uses and extends the Allometric Trophic Network^52,53^ (ATN) model by including plankton subsidy. Both approaches found that the intertidal food web is highly robust to the loss of all harvested species, as no secondary extinctions were observed after removing all harvested species (Fig. 2). We also found that relatively more secondary extinctions occurred with random deletion sequences than with the loss of all harvested species (Fig. 2). The robustness of the intertidal food web was further demonstrated by the sequential deletion of the most connected species, which showed that over 30% of those species must be removed before any secondary extinctions occur (Fig. 2). As expected from previous work^42^, the loss of supporting-basal species produced the most secondary extinctions (Fig. 2). Both approaches showed similar trends (Fig. 2A,B), but our dynamic approach presented relatively lower food web robustness (Supplementary Fig. S2).

Although the plankton node (“trophic species”) was directly connected only to filter-feeders (Fig. 1B), it proved to be the most important in the supporting-basal deletion sequence for both static and dynamic approaches, as its removal caused 15 and 29 secondary extinctions, respectively. Both approaches found that all filter-feeders (n = 15) went secondarily extinct when plankton was removed because filter-feeders are specialist consumers of plankton. The dynamic approach also found that carnivores (n = 6) and top-predators (n = 7) went secondarily extinct when plankton was removed, even when alternative resources persisted. This means that the abundance of those alternative resources was not enough to ensure the persistence of carnivores and top-predators. Note that the presence of alternative resources for carnivores and top-predators is sufficient for them to persist under the static approach. The species that went extinct with the removal of plankton with the dynamic approach included not only the sessile filter-feeders, but also four harvested species important for the fisheries: the Chilean muricid whelk *Concholepas concholepas*, the giant barnacle *Austromegabalanus pssitacus* (also went extinct with the static approach), the sea squirt *Pyura chilensis* (also went extinct with the static approach) and the whelk *Acanthina monodon*. These results suggest that while the intertidal food web is robust to harvest-driven extinctions, it can be sensitive to a drastic decrease in plankton productivity.

### Effects of artisanal fisheries on food web dynamics

**Figure 3:**
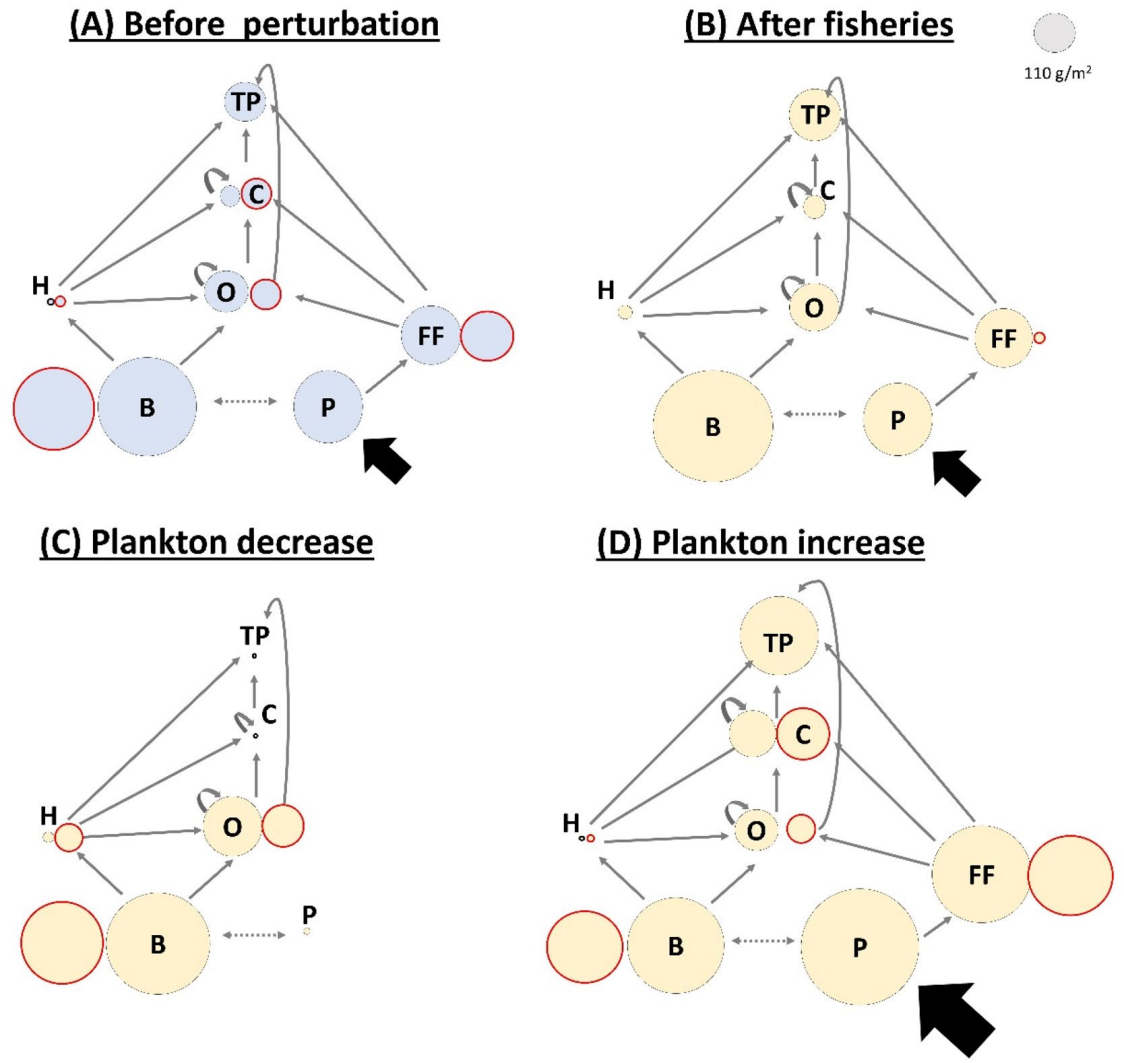
Diagram illustrating the effect of fisheries (**B**) and the effect of perturbing plankton subsidy (**C** and **D**) on food web dynamics. Nodes represent the total biomass of each trophic level before (**A**) and after (**B**) reducing in − 100% the biomass of all harvested species, and after decreasing (− 100%, **C**) or increasing (+ 100%, **D**) the plankton subsidy (with respect of its basal value = 7355 g/m^2^). Each trophic level is indicated by TP: top predators, C: carnivores, O: omnivores, H: herbivores, FF: filter-feeders, B: basal species, and P: plankton. Red and black outlined nodes represent the biomass of harvested and non-harvested species, respectively. Solid black, solid grey, and dashed grey arrows represent the plankton subsidy, trophic interactions, and competitive interactions, respectively. Note that the ATN model explicitly models competition only between basal species, while competition between consumers emerges from the depletion of shared resources.

We assessed the effects of fisheries on the biomass of every species in the food web using our extension of the ATN model (see “Methods”). Figure 3A,B summarize with a simplified diagram the results shown in Supplementary Fig. S3. We simulated three fishing scenarios, where we applied exploitation rates needed to decrease the biomass of all 22 harvested species in − 50%, − 80%, and − 100% of their original biomass (see F_max_ in Supplementary Table S3). These three fishing scenarios allowed us to simulate approximately well managed fisheries (which removes between 40 and 60% of biomass stock^5^), an overexploitation scenario (which removes 80%) and nearly extinction scenario, respectively. We found that basal species required much lower exploitation rate to decrease their biomass than filter-feeders, herbivores, and other consumers (Supplementary Table S3, see “Discussion” for an explanation). Harvested basal species went extinct with an extraction above 0.3% of their available biomass, while harvested consumers went extinct with an extraction above 90% of their available biomass.

The decrease in biomass of harvested species increases the biomass of most non-harvested species, especially of basal and herbivorous species (compare Fig. 3A,B). In fact, more than 80% of non-harvested species increased their biomass by 5–25% after fishing reduced in − 50% to − 100% the original biomass of harvested species (Supplementary Fig. S3). This biomass increase of non-harvested species helps to explain why we found no secondary extinctions using the ATN model, and it is caused by two mechanisms: i) decreasing the biomass of harvested species that are consumers reduces the predation intensity on their resources (note that fisheries harvest more species in higher than lower trophic levels, compare Supplementary Fig. 1B,C), and (ii) decreasing the biomass of harvested basal species reduces their competitive effects on the non-harvested basal species, allowing them to grow (Supplementary Fig. S4).

The positive effect of artisanal fisheries on the biomass of non-harvested species was qualitatively similar across the different fishing scenarios, becoming larger with an increase in fishing intensity (Supplementary Fig. S3). The exceptions were top predators, which had opposite responses between the weakest and strongest fishing scenarios. A − 50% reduction of the biomass of all harvested species slightly decreased the biomass of non-harvested top predators, while reductions of − 80% and − 100% slightly increased their biomass. This suggests that artisanal fisheries negatively impact top predators by extracting their prey species but, when the exploitation rates are stronger, the indirect positive effects of fisheries on the biomass of the non-harvested species become strong enough to dampen those effects.

### Effects of plankton-subsidy alteration on food web dynamics

We considered an externally controlled subsidy of plankton productivity. We both decreased (− 50%, − 80%, − 100%, Fig. 3C) and increased (+ 50%, + 80%, + 100%, Fig. 3D) the plankton subsidy with respect to the original plankton subsidy biomass to simulate the alteration of plankton productivity expected as a response of climate change (see the arguments supporting our perturbation levels in “Methods”). All biomass changes can be found in Supplementary Fig. S5. Both decreasing (Fig. 3C) and increasing (Fig. 3D) plankton subsidy can deeply alter food web dynamics. The magnitude of change in plankton subsidy affected the food web patterns shown in Fig. 3C,D only quantitatively, becoming more intense with an increasing alteration of the plankton subsidy. Decreasing plankton subsidy had larger impacts on the species biomasses than increasing plankton subsidy in the same magnitude, even causing species extinctions (i.e., − 1 in Supplementary Fig. S5E) when the subsidy was removed (i.e., − 100%). The number of total extinctions that occurred after completely removing the plankton subsidy was 29 species, highlighting the bottom-up propagation of effects through the food web (Supplementary Fig. S5E).

A drastic decrease in plankton subsidy (− 100%) resulted in the extinction of all filter-feeder species (specialist consumers of plankton) and decreased the biomass of carnivores and top predators by 99% (compare Fig. 3A,C). The biomass reduction in carnivores and top predators, in turn, released predation pressure on omnivores and herbivores, which consequently increased their biomass by 30% and 110%, respectively. The increased biomass of herbivores and omnivores, in turn, increased consumption pressure on basal species, but we found that the biomass of basal species is almost invariant with a slight increase of 4% (Fig. 3C). This suggests that the reduction in plankton subsidy positively affects basal species by releasing pressure on the community level carrying capacity (see “Methods”). Conversely, a + 100% increase in plankton subsidy increased the total biomass of filters, carnivores, and top predators by 76%, 107%, and 105%, respectively (compare Fig. 3A,D). As a consequence, the increased predation pressure from higher trophic levels decreased the total biomass of herbivores by 20%, but the total biomass of omnivores and basal species almost did not change with a slight decrease of 2% and 3%, respectively. Carnivore species were the most vulnerable to the reduction of plankton productivity, going extinct with a reduction of − 80% in plankton subsidy (Supplementary Fig. S5C), followed by filter-feeders and top predators, which went extinct with a − 100% of subsidy reduction (Supplementary Fig. S5E). Regarding harvested species, 18% of them strongly decreased their biomass when plankton subsidy decreased, while 81% of them slightly decreased their biomass when plankton subsidy increased (compare Supplementary Fig. S5A,C,E with S5B,D,F).

### Interacting effects of fisheries and plankton-subsidy alteration on food web dynamics

**Figure 4:**
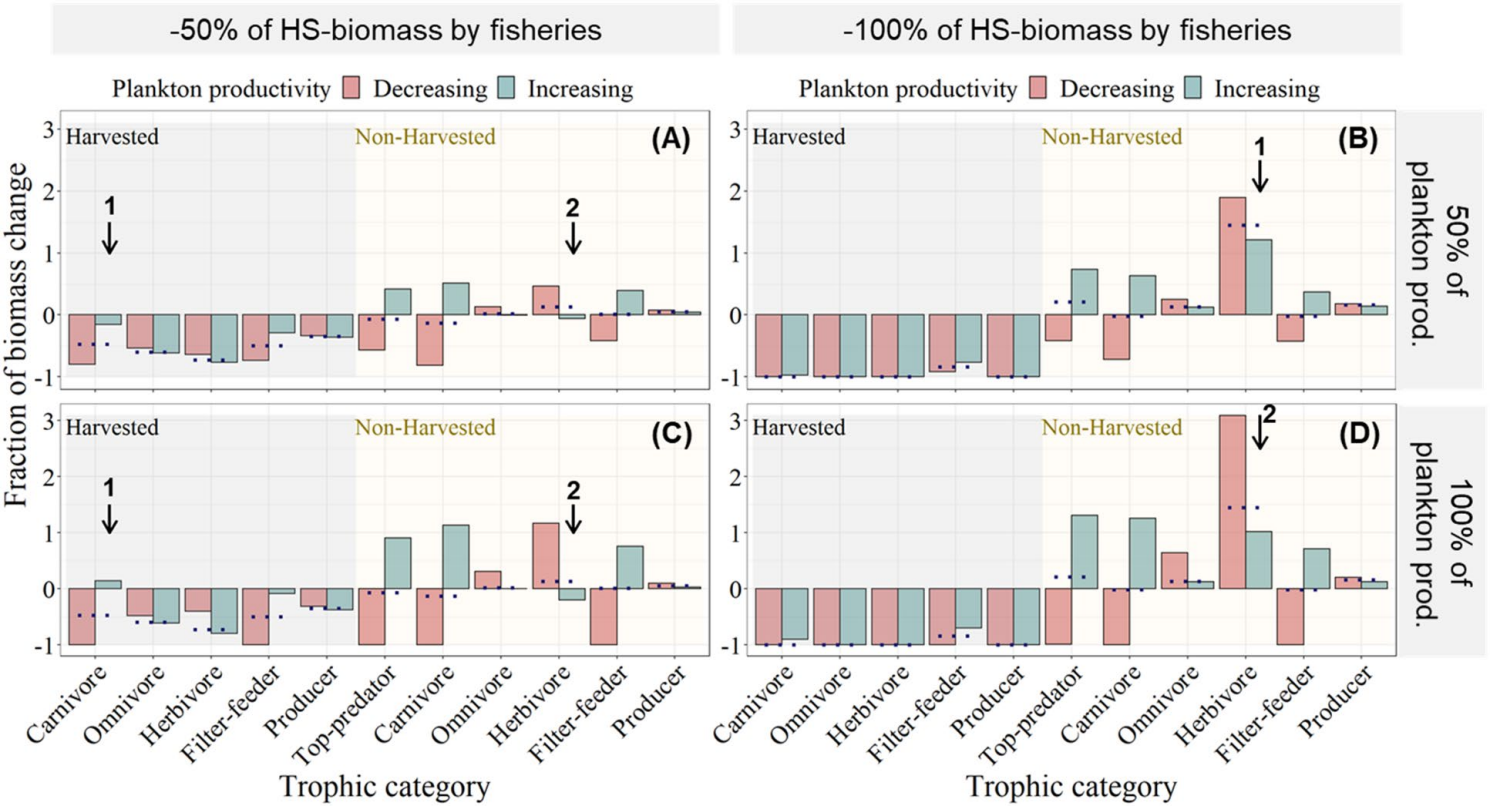
Combined effects of artisanal fisheries and alterations in plankton productivity on food web dynamics. Fraction of total biomass change (y-axis) of each trophic category (x-axis) after decreasing (red bars) and increasing (blue bars) the plankton productivity (plankton prod.) in 50% (**A** and **B**) and 100% (**C** and **D**), and after decreasing the biomass of all harvested species (HS) in a 50% (**A** and **C**) and in a 100% (**B** and **D**). The grey and yellow shading represent the biomass change of harvested and non-harvested species, respectively. The arrows highlight the most remarkable changes between the two levels of plankton subsidy perturbation and the two levels of fishing. The dotted lines represent the independent effect of fishing (i.e., without plankton subsidy perturbation) on the biomass of each trophic category as a reference point.

We evaluated the combined effects of the biomass extraction by fisheries and the alteration of plankton subsidy on the food web dynamics using a full factorial design that maintains the same fishing (− 50%, − 80%, − 100% of the original biomass of harvested species) and plankton subsidy (− 50%, − 80%, − 100%, + 50%, + 80%, + 100% of the basal subsidy of plankton biomass) levels used in each of the last two sections. We found that regardless of the fishing scenario, all non-harvested trophic levels persisted when the plankton subsidy increased or decreased (Fig. 4A,B) by ± 50%. Conversely, when the plankton subsidy decreased in − 80%, carnivores went extinct under all fishing scenarios (compare Supplementary Fig. S6C,D with S6A,B,E,F) as well as the top predators and filter-feeders when the plankton subsidy decreased in − 100% (Fig. 4C,D).

The level of plankton subsidy affected the impact of fishing on the biomass of harvested species. Decreasing plankton subsidy decreased the biomass of harvested carnivores and filter-feeders, intensifying the negative effect of fishing on their biomasses (see the two black arrows labeled with a number 1 pointing down to such results for “Harvested Carnivores” in panels A and C of Fig. 4). The reverse occurred when increasing plankton subsidy, which dampened the effect of fishing on the biomass of harvested carnivores and filter-feeders. Interestingly, decreasing plankton subsidy also increased the biomass of harvested and non-harvested omnivores and herbivores (see results of “Effects of artisanal fisheries on food web dynamics” section), which therefore dampened slightly the negative effect of fisheries on harvested omnivores and herbivores (Fig. 4A,C).

Fisheries also affected the impacts of perturbing plankton subsidy on species biomasses. Increasing fishing increased the biomass of non-harvested species (see results of “Results” section) and, therefore, dampened the negative effects of altering plankton subsidy on the biomass of these species while intensifying the positive effects of altering plankton subsidy in those species (compare panels A and C with B and D of Fig. 4). Specifically, fisheries reversed the negative effect of increasing plankton subsidy on the biomass of non-harvested herbivores (see 4 black arrows labeled with a number 2 pointing down to such result in panels A–C of Fig. 4).

## Discussion

Fisheries are commonly associated with negative impacts on ecosystems, especially on the food web structure, total biomass, and species recovery^54–56^. The present study shows that these effects are not necessarily general at a food web scale. We found that in a rocky-shore intertidal food web, the simulated extinction of all harvested species caused no secondary extinctions. This despite artisanal fisheries harvesting on more than 20% of the food web species, which are also highly connected species. In addition, we found that this food web was highly vulnerable to the decrease of plankton productivity, which is one of the outcomes expected to happen as consequence of climate change^24,30,33,34^. Finally, we found that artisanal fisheries might contribute to dampening the negative consequences of the decrease in plankton productivity by increasing the biomass of non-harvested species. In the following paragraphs, we expand on these results and contextualize them with prior literature.

The impact on the food web structure of all harvested species going extinct (i.e., food web shortened and connectance decreased, Supplementary Table S2) caused no secondary extinctions. This suggests that harvested species are embedded in redundant^57^ trophic interactions (see below). Moreover, our dynamic approach found that fisheries reduced both the predation pressure of harvested consumers on non-harvested resources and the interspecific competition between harvested and non-harvested basal species, which explains the high robustness found with the dynamic approach. In addition, the high robustness of the food web suggests that the exploitative competition between artisanal fisheries with harvested and non-harvested species for common resources (Supplementary Fig. S1B) might be weak, as consumers have wider diets that can buffer the loss of harvested species. These results, however, do not imply that the extinction of all harvested species would not impact the food web. Over-exploiting the harvested species to extinction is per se a negative effect. Moreover, the socio-economic system in which fishers are embedded will be directly impacted^58^ if resource management by local TURFs fails and drive the harvested species extinct. Fishers would need to harvest on new species as alternative resources to maintain their livelihood, which will impact the food web in ways we did not explore here. In our analysis, the loss of harvested species caused the loss of an important amount of redundant links (Table S2), suggesting that the resulting food web contains a greater predominance of functional than redundant links and, therefore, less robust to further species extinctions^59^.

Although our results are surprising, they are not unreasonable. Previous studies have shown that food webs can be very robust to realistic extinction sequences. In the Serengeti food web, the extinction of species based on their degree of endangerment according to the IUCN criteria, caused no secondary extinctions^60^. Similarly, 50 pelagic food webs from small lakes and ponds in the Adirondack Mountains of New York State, USA, were remarkably robust to the extinction of species in order of their pH tolerance^61^. In our case, the high redundancy of trophic interactions is explained by its high levels of omnivory^14^, generalist consumers^62^, and a high proportion of transient and weak links^63^, which seems to be common in Chilean rocky intertidal ecosystems^13,62,63^. These attributes confer food webs alternative routes of energy and stability^49,64^.

Our results also highlighted the vulnerability of basal species to fishing, with basal species going extinct with an extraction above 0.3% of their available biomass. This is because harvested basal species are consumed by 2.5 more species than harvested consumers, and their intrinsic growth rate is 3 times lower than that of non-harvested basal species as they are the macroalgae that have the largest body size. Among the harvested basal species is kelp, which plays an important ecological and economical role. Kelp provides habitat structure and shelter to many species^12^ and via this non-trophic interaction, it promotes the biodiversity of coastal ecosystems^14,65^. By considering only trophic interactions, however, we found that the extinction of all harvested species (including kelps) did not produce secondary extinctions. This contradiction suggests that the non-trophic interactions of kelps with other species might be key to understand the impacts of fishing. Therefore, we should interpret the impacts of kelp fishery carefully. Kelps commercial value is high, with Chile being one of the main exploiters of kelp natural populations^66^. Kelp extraction in Chile is managed but hardly supervised^66^. Therefore, kelp’s high demand, high value, and low control, leave these algae prone to illegal fishing. In this context, we highlight the urgency of increasing supervision of kelp fisheries and enforcing their compliance with management plans.

In the rocky intertidal ecosystems we studied in this work, artisanal fishermen obtain their resources through hand-picking and use them for self-subsistence^6^. Therefore, resource availability in intertidal ecosystems plays an important role for the poorest fishermen. Artisanal fishers with more means apply most of their fishing gears (e.g., diving, spearing, and pot trapping) in subtidal-shallow ecosystems^6^, from where they harvest ~ 20% of species in the food web (Supplementary Fig. S7A). Therefore, we repeated our static extinction analysis in the shallow-subtidal marine food web and found that, as in the intertidal food web, the subtidal food web is robust to the loss of all harvested species (Supplementary Fig. S7B). This suggests that similar mechanisms explaining the high food web robustness of the intertidal ecosystem against the extinction of harvested species, explain the high food web robustness of the subtidal. Moreover, as in the intertidal food web, we found that plankton was the most important group (node) for the persistence of the subtidal food web. Analyzing the effect of artisanal fisheries on the subtidal food web with a dynamic approach seems an important next step to understand how anthropogenic activities as well as bottom-up and top-down forces affect coastal food webs.

A concerning effect of climate change is the alteration of plankton productivity. This can be caused by the physicochemical changes in coastal waters triggered by warmer waters^24^ and by an intensification of upwelling-favorable winds^35,67^, accompanied with a decrease (or increase) of nutrients given by an intensification of the warm (or cold) phase of ENSO^68–70^. The importance of plankton is well-known as the energy supply of food webs, as well as essential for sustaining fisheries^32^. We found that plankton is the most important food web component for species persistence. Plankton is consumed by filter-feeders and any alteration of plankton subsidy affects the biomass of all the species in the food web. On the one hand, a decrease in plankton subsidy caused the intertidal food web to shorten, with strong impacts on fisheries because of the biomass reduction of harvested species. Similar results were found when climate change effects were simulated as an increase of biological rates^22,71^ caused by the increase in temperature, which suggests that our results will be magnified if we were to consider the alteration of biological rates. In addition, a reduction in plankton productivity may reduce the recruitment of species (as plankton composition also include larvae of several species^72^), which might cause more secondary extinctions than we found here. On the other hand, an increase in plankton subsidy negatively impacted the biomass of a higher number of species but in smaller magnitude than the decrease of plankton subsidy. Moreover, an enrichment of nutrients can increase the arrival of new species^73^ or the recurrence of harmful algal blooms with a devastating effect on local food webs^74^. Thus, if we consider these factors, we would expect an intensification in the negative consequences observed in this study.

We found that the effects of the changes in coastal productivity and artisanal fisheries on the dynamics of the intertidal food web interact, which reinforces the call made by previous studies^75–77^ that more research is needed to understand the interaction of several environmental stressors on ecosystems. In particular, we found that fisheries dampened the negative impacts of decreasing plankton productivity on non-harvested species, while the decline in plankton productivity increased the sensitivity of harvested species to fishing. This positive effect of fisheries on non-harvested species is explained by the increase in the biomass of non-harvested species caused by fisheries harvesting more on those species’ predators (Supplementary Fig. S1C) than preys (Supplementary Fig. S1B), and by fisheries reducing the interspecific competition between basal species through decreasing the biomass of the basal species they harvest (see Supplementary Fig. S4). Our results are consistent with previous work^78^ showing that human-gatherers enhance the species persistence of coastal marine ecosystem in the North Pacific. This suggests that, at least in the intertidal food web studied here, small-scale artisanal fisheries play a similar role as human-gatherers in the North Pacific, that is, as keystone species to the non-harvested species of the food web.

Limitations of our research mostly consist of factors not included in our modeling approach. For example, as explained above, our model does not include facilitation or mutualistic interactions (nor direct competition among consumers), which can be critical for the dynamics of food webs^79–81^. Other factors not considered in this study are local spatial features such as the enclosed bay with internal circulation and larval retention^82^ as well as upwelling zones^83^, which can affect species recruitment and affect our results. Similarly, temporal variability and other stochasticity sources associated with global change (e.g., invasive species, pathogens spread, habitat degradation, and several climatic stressors^1,2^) can also change the relative importance of species in food webs, making an open-system approach^48^ to ecological networks an important next step. For example, the extinction of all harvested species might release several ecological niches and, consequently, fisheries might increase species invasion. Moreover, as invasive species are characterized by generalist foraging habits and lacking predators^84^, we might find negative consequences in the abundance of local non-harvested species. Thus, our results should be interpreted cautiously, and the positive effects of fisheries do not mean that fisheries can indiscriminately exploit these ecosystems. These effects might depend on adaptive prey-switching behavior (mechanism not considered here), allowing them to use alternative or new resources in response to changes in abundances of other species in the community, and with that rewire food web^85^ and stabilize populations dynamics^86^.

## Methods

### Food web description and the relative importance of harvested species to the food web structure

We studied a well-resolved food web of the intertidal rocky shore communities of the central coast of Chile^12^, which is harvested exclusively by small scale artisanal fisheries^11^. The web represents all species that are found to co-occur on wave exposed rocky platforms of central Chile, from the very low to the highest intertidal and is composed of 107 species (including a fisheries node), with 44% of its species corresponding to primary producers, 53% to invertebrates, and 3% to endotherm vertebrates. In the food web, we consider as basal level all species of benthic primary producers plus plankton (phytoplankton + zooplankton, single node). Therefore, we represented filter-feeders (sessile filter-feeders + porcenallidae crabs) as specialist consumers of plankton and not as basal species (see detailed description of the food web in Supplementary Material).

Species harvested by artisanal fisheries were identified using information from the Chilean national fishing service (www.sernapesca.cl) and previous work^13^. A high diversity of species distributed across all trophic levels are harvested by artisanal fisheries (red nodes in Fig. 1), including numerous species of macroalgae (n = 7), filter-feeders (n = 2), herbivores (n = 1), omnivorous (n = 10), and carnivores (n = 2), totaling 22 species. Using the static approach (without population dynamics, see next subsection), we compared the structure of the food web with and without the harvested species to the distribution of 1000 food web structures produced by randomly removing the same amount of harvested species (see more details about this method in supplementary materials).

### Static and dynamic approaches for evaluating food web robustness

The static approach stems from network theory and analyzes the impacts of structural changes on food webs represented by nodes (species) and links (interactions) that connect nodes, but ignores interaction strengths and population dynamics of interacting species^38^. In this approach, a non-basal species is considered extinct after a perturbation (defined here as a secondary extinction) if all its resource species (food) go extinct. Basal species are assumed to be autotrophs or otherwise obtain resources from outside the modeled web (e.g., through subsidies from other ecosystems) and, therefore, do not experience extinctions unless directly removed (defined here as a primary extinctions). Thus, the static approach only considers extinctions produced by direct bottom-up effects. A dynamic approach considers not only the structure and intensity of interactions in a food web, but also the changes in species abundances through time and the indirect and dynamic effects that these changes have on the abundances of other species^39–41,48,49^. A species is then considered to be secondarily extinct when its abundance drops below a threshold as a consequence of its population losses being higher than its population gains. Therefore, a dynamic approach can take into account both bottom-up and top-down effects of perturbations on the system, and both forces can cause secondary extinctions^39^. We use both the static network-based approach and a dynamic approach based on energy-transfer (see dynamic model below) to evaluate the impacts of artisanal fisheries and changes in primary productivity on the intertidal food web.

### The dynamic model

The Allometric Trophic Network (ATN) model^52,53^ consists of two basic sets of equations, one set describing the biomass changes of primary producers (Eq. 1) and the other describing that of consumers (Eq. 2), where ***B*** is the biomass vector with the biomasses of every species population in the food web and ${B}_{i}$ is the biomass of the population of species *i*, as follows:

1 $$\frac{{dB_{i} }}{{dt^{\prime } }} = \overbrace {{r_{i} B_{i} G_{i} \left( {\varvec{B}} \right)}}^{Autotrophic\,growth\, gain} - \overbrace {{\mathop \sum \limits_{j} \frac{{x_{j} y_{ji} B_{j} F_{ji} \left( {\varvec{B}} \right)}}{{e_{ji} }} }}^{Herbivory\,loss} - \overbrace {{F_{\max i} B_{i} }}^{Fisheries\,loss} .$$

2 $$\frac{{dB_{i} }}{{dt^{\prime } }} = \overbrace {{f_{a} x_{i} B_{i} \mathop \sum \limits_{j} y_{ij} F_{ij} \left( B \right)}}^{Resources\,consumption\, gain} - \overbrace {{f_{m} x_{i} B_{i} }}^{Maintenance\,loss} - \overbrace {{\mathop \sum \limits_{j} \frac{{x_{j} y_{ji} B_{j} F_{ji} \left( {\varvec{B}} \right)}}{{e_{ji} }}}}^{Predation\,loss} - \overbrace {{F_{\max i} B_{i} }}^{Fisheries\,loss}.$$

The biomass of producer *i* changes according to the balance of autotrophic growth gain and losses due to predation by *j*. The net autotrophic growth is determined by the logistic growth function $G_{i} \left( B \right) = 1 {-}\left( {\sum\nolimits_{j = producers} {c_{ij} B_{j} } } \right) /K$, where *r*_*i*_ is the intrinsic growth rate of producer *i*, ${c}_{ij}$ is the inter-specific competition coefficient between producer *i* and *j*, and *K* is the total carrying capacity of primary producers in the system. The biomass loss of producer *i* by herbivory (caused by herbivores or omnivores) increases with the mass-specific metabolic (*x*_*j*_) and attack (*y*_*j*_) rates of consumer *i*, and decreases with the assimilation efficiency of consumer *i* for resource *j* (${e}_{ij}$). The consumers’ population dynamics (Eq. 2) depend on their mass-specific metabolic rates (*x*_*j*_) and on the balance between biomass gains by resource consumption, biomass loss by metabolic maintenance, and biomass loss to predation. From the total amount of resources ingested by the consumer population *i,* $\sum_{j}{y}_{ij}{F}_{ij}\left({\varvec{B}}\right)$, only a fraction *f*_*a*_ is assimilated into consumer available energy for maintenance and biomass growth. The functional response *F*_*ij*_(***B***) determines the consumption rate of each consumer *i* for each resource *j*, defined by:

3 $${F}_{ij}\left({\varvec{B}}\right)= \frac{{\omega }_{ij}{B}_{j}^{q}}{{B0}_{ij}^{q}+ {d}_{i}{B}_{i}{B0}_{ij}+ {\sum }_{l=resources}{\omega }_{il}{B}_{l}^{q}}$$

where ${\omega }_{ij}$ is the relative preference of consumer *i* for resource *j*, *q* controls the shape of Eq. (3) which becomes an intermediate functional response between type II and type III when *q* = 1.2^87^. ${B0}_{ij}$ is the biomass of resource *j* at which the consumer *i* achieves half of its maximum consumption rate on resource *j*, and ${d}_{i}$ is the intra-specific interference of consumer *i* when it forages resource *j*. In Eq. (2), *f*_*m*_ defines the fraction of the consumer biomass that is respired for maintenance of basal metabolism. *F*_*max*_ defines the fraction of biomass *i* that is removed by small-scale artisanal fisheries. In the case of non-harvested species *F*_*max*_ = 0.

The biological rates of production, R, metabolism, X, and maximum consumption, Y, follow a negative power law with the species body size (M), with an exponent − 1.4^88^:

4 $$R_{P} = a_{r} M_{P}^{ - 0.25}$$

5 $$X_{C} = a_{x} M_{C}^{ - 0.25}$$

6 $$Y_{c} = a_{y} M_{C}^{ - 0.25}$$

where a_r_, a_x,_ and a_y_ are allometric constants and the subscripts P and C denote producers and consumers, respectively. The timescale to examine the dynamics of the system is defined based on the primary producer with the highest mass-specific growth rate (reference species). The mass-specific growth rate and the metabolic rate of each species were normalized by the growth rate of the reference species, and the maximum consumption rate was normalized by each species’ metabolic rate^88^. These normalizations translate to the following expressions of intrinsic growth rate (*r*_*i*_), metabolic rate (*x*_*i*_), and maximum consumption rate (*y*_*i*_) of each species *i*:

7 $${\mathrm{r}}_{\mathrm{i}}=\frac{{R}_{P}}{{R}_{Pref}}=1 {\left(\frac{{M}_{P}}{{M}_{Pref} }\right)}^{-0.25}$$

8 $${\mathrm{x}}_{\mathrm{i}}=\frac{{X}_{C}}{{R}_{Pref}}=\frac{{a}_{x}}{{a}_{r}} {\left(\frac{{M}_{C}}{{M}_{Pref} }\right)}^{-0.25}$$

9 $${\mathrm{y}}_{\mathrm{i}}=\frac{{Y}_{C}}{{X}_{C}}=\frac{{a}_{y}}{{a}_{x}}$$

Since most benthic marine communities are characterized by the presence of sessile filter-feeders at the bottom, these communities are heavily ‘subsidized’ by the pelagic phytoplankton, which is captured by filter-feeders and transferred up the benthic food web^89^. Phytoplankton dynamics is thought to vary primarily due to ‘external processes’ (e.g. water advection, nutrient loadings, etc.), including climate fluctuations^34^. To account for this phenomenon, our implementation of the ATN model assumes that the intertidal community is permanently subsidized by plankton biomass. Therefore, we modeled plankton dynamic as a primary producer (Eq. 1) and following^75,76^ we incorporated a constant subsidy *s* into the plankton dynamics as:

10 $$\frac{dBi}{{dt^{\prime } }} = d_{local} + s,$$

where *d*_*local*_ represents plankton local dynamics (i.e., right hand of Eq. 1).

To our knowledge, the model we developed here is the largest food web dynamic model ever empirically parameterized using the ATN framework. See model parametrization in the supplementary material and parameters values in Supplementary Table S4. Moreover, the added realism of plankton subsidy allows us to simulate the effect of climate change as the alteration of plankton subsidy. Our dynamic analysis per se is a sensitivity analysis of the two new factors we added to the ATN model, that is, plankton subsidy and added mortality due to harvesting. This is because our entire dynamic analysis is a full factorial simulation design with three levels of harvesting and six levels of plankton subsidy that extensively cover the parameter space of those two factors (see last subsection “Interacting effects of fisheries and plankton-subsidy alteration on food web dynamics”). Previous work shows that the ATN model is capable of describing community dynamics remarkably well^88,90–93^ and that it is robust to parametrization^53^. Part of the analytical power of the ATN approach is that ecological interactions are reasonably parameterized with species body masses, allowing researchers to focus their on other model aspects^50^.

### Food web robustness to species extinctions

Using the static and dynamic approaches (see the model above), we evaluated the food web robustness to species extinctions using four deletion sequences (see below). We chose these deletion sequences to evaluate the secondary extinctions caused by over-fishing, and to compare those secondary extinctions to the ones caused by random sequences and by the most detrimental deletion sequences described for food webs (“most-connected” and “supporting-basal”, see below). First, we evaluated the potential secondary extinctions caused by over-fishing through removing the harvested species in descending order of total catch amount (hereafter “harvesting” deletion sequence), according to the Chilean national fishing service (www.sernapesca.cl). Second, we performed three additional deletion sequences: (1) randomly (hereafter “random” deletion sequence), (2) from the most to the least connected species (hereafter “most-connected” deletion sequence^38,41^), and (3) from the most connected species that trophically support highly connected species to the least connected species supporting low connected species^42^. This last sequence causes the fastest route of collapse^42^ by first deleting the basal species that support most of the species in the food web (hereafter “supporting-basal” deletion sequence). For the harvesting deletion sequence, species were removed until all the harvested species were deleted, while for all other sequences the procedure was repeated until all species were removed (including the harvested species). In the case of species with an equal number of interactions, the removed species was chosen at random^40^.

We use the R_50_ index^38^ to compare the food web robustness across the different patterns of species deletion, except the “harvesting” deletion sequence. For the harvesting deletion sequence, only the number of secondary extinctions was registered. The R_50_ index represents the proportion of species that have to be removed to cause the extinction of 50% of the species in the network (including primary and secondary extinctions). The highest and lowest possible values of R_50_ are 0.5 and 1/S, respectively (S is the number of species in the network), which are reached when no secondary extinctions are caused by species deletions and when only one primary species deletion is needed to cause the extinction of 50% of species, respectively. Thus, larger values of *R*_*50*_ mean higher robustness. The static approach was simulated using the R package *NetworkExtinction*^94^, while the dynamical model was simulated using ODE45 in MATLAB.

For the dynamic approach, we first ran the dynamic model for 3650 time steps which corresponds to 10 years, and ensures that the food web reached a dynamic equilibrium. Then, we started the removal simulations. In each removal step, the number of extinct species was recorded after 10 years, when the system had reached, again, a steady state. A species was considered extinct if its biomass diminished to less than 10^–6^^95^. Note that in all deletion sequences we removed the nodes from the food web, so in the harvesting deletion sequence the F_max_ parameter in the ATN model is zero to all harvested species.

### Effects of artisanal fisheries on food web dynamics

We assessed the effects of artisanal fisheries on food web dynamics by simulating simultaneous fishing on all harvested species and assessing the subsequent effects on the biomass of all species in the food web. We simulated three fishing scenarios, where we applied exploitation rates needed to decrease the biomass of all harvested species in − 50%, − 80%, and − 100% (see F_max_ in Supplementary Table S3) with respect to their original biomass before a fishing scenario. Note that basal species required much lower exploitation rate to decrease their biomass (see “Discussion”) than filter-feeders, herbivores, and other consumers, which means that harvested basal species were the most sensitive species to fishing. Note also that a biomass decrease of 100% does not necessarily mean that the harvested species go extinct, because the biomass available to be removed by fishing is the biomass that was produced a time step earlier (i.e., fishing exploitation is simulated as part of the population dynamics of harvested species, see Eqs. 1 and 2). These three fishing scenarios allowed us to simulate an approximately well managed fisheries (which removes between 40 and 60% of biomass stock^5^), an overexploitation scenario (which removes 80%) and nearly extinction scenario, which allowed us to assess overall stability if all harvested species go extinct. For each fishing scenario, we first ran the model for 10 years (3650 time steps) to ensure that the system reached a dynamic equilibrium. Then, we applied the biomass removal (*F*_maxi_*B*_i_ in Eqs. 1 and 2) at each time step in the model to all harvested species simultaneously and we ran the food web dynamics for another 3650 time steps to reach post perturbation equilibrium, when final biomasses were considered “after perturbation” state.

In each treatment and for each species *i*, we evaluated the effect of the simulated scenario as:

11 $$Biomass \,change_{i}=\left[\left(\frac{{after \,perturbation_{i}}}{{before \,perturbation_{i}}}\right)-1\right]*100$$

### Effects of plankton subsidy alteration on food web dynamics

We assume that the plankton dynamics is subsidized by an external source. This subsidy to plankton is considered to be controlled by advective processes, unaffected by local benthic consumption. This represents well the situation of most marine benthic ecosystems^89^. We simulated both a decrease and an increase in plankton subsidy, because both long-term increased and decreased productivity has been documented to occur in the Humboldt Ecosystem^34^. We used three different perturbation intensities, decreasing (−) or increasing (+) basal subsidy in ± 50%, ± 80% and ± 100%. Note that a − 100% in the basal plankton subsidy does not translate into plankton extinction (Fig. 3C). A variation of ± 50% of the basal subsidy is in the order of natural seasonal variability of net primary productivity in central Chile^31^. Therefore, we assumed that a variation above + 50% and below − 50% simulates the effects of extreme changes of plankton subsidy due to climate change. In addition, those magnitudes allow comparable perturbation intensities to those used to assess the impacts of fisheries on the food web dynamics (see previous section). In each scenario, we first ran the model for 3650 time steps to ensure that the system reached a dynamic equilibrium, and the final species biomasses obtained were considered “before perturbation” state. Then, we reduced/increased plankton subsidy at each time step and ran the model for another 3650 time steps to reach post perturbation equilibrium. The final biomasses were considered “*after perturbation”* state. Changes in biomass were expressed as shown in Eq. (11).

### Interacting effects of fisheries and plankton-subsidy alteration on food web dynamics

To evaluate combined effects of fisheries and perturbation in plankton subsidy, we simulated fishing on all harvested species and simultaneously altered plankton subsidy. We used the three fisheries scenarios (i.e., − 50%, − 80% and − 100% biomass removed) and crossed these scenarios with each of the six productivity scenarios (i.e., + 50%, + 80% and + 100%, − 50%, − 80% and − 100% of the basal plankton subsidy biomass). In each treatment, we first ran the model for 3650 time steps and the final species biomasses obtained were considered “*before perturbation”* state. Then, we applied a given plankton subsidy scenario, and at the same time, we started the fishing simulations. Changes in biomass were expressed as shown in Eq. (11).

## Supplementary Information

Supplementary Information

## Data Availability

Simulation code and the Chilean intertidal data will be available upon acceptance at the repository https://github.com/fsvaldovinos/Chilean_Fisheries. The Chilean intertidal food web and species body sizes can also be found in ^12^.
