# Supplementary material for: Alteration of coastal productivity and artisanal fisheries interact to affect a marine food web
Source: Sci Rep. 2021 Jan 19;11:1765. doi: 10.1038/s41598-021-81392-4 (PMC7815714; doi:10.1038/s41598-021-81392-4)
Supplement: Supplementary file 1 — Supplementary Information [file 41598_2021_81392_MOESM1_ESM.docx]

Alteration of coastal productivity and artisanal fisheries interact to affect a marine food web

M. Isidora Ávila-Thieme, Derek Corcoran, Alejandro Pérez-Matus, Evie A. Wieters, Sergio A. Navarrete, Pablo A. Marquet & Fernanda S. Valdovinos

### **Food web description**

The intertidal rocky-shore food web is influenced by the highly productive Humboldt Current System^1^ (HCS). Is composed of 107 species (including fisheries node). A total of 1381 consumption type interactions have been documented in the web, with approximately linear increase in species degree (number of interactions per node) and species rank^2^, and connectance of 0.12. We consider as basal level all species of benthic primary producers (e.g. algae) plus plankton (phytoplankton + zooplankton, single node). Therefore, we represented filter-feeders (sessile filter-feeders + porcenallidae crabs) as specialist consumers of plankton and not as a basal species. Benthic diatoms were considered as an independent node, separate from plankton. Detailed methods and general description of food web attributes, as well as patterns of spatial variability can be found in [2–4].

### **Relative importance of harvested species for the food web structure**

Using the static approach (without population dynamics), we compared the structure of the food web with and without the harvested species to the distribution of 1000 food web structures produced by randomly removing the same amount of harvested species. This comparison used: number of species (S), number of trophic links (L), connectance (L/S^2^), number of omnivores, and the mean short-weighted trophic level (MeanSWTL^5^). We calculated all these structural metrics using cheddar package in R. After removing all harvested species using our static approach, the food web resulted in lower link density (*L*/*S*, where *L* and *S* are numbers of trophic interactions and species, respectively) and connectance (C = *L*/*S* ^2^ ) than removing the same number of species at random (Table S2). This decrease in connectance is counter-intuitive because the pure removal of species should increase connectance based on its mathematical definition. Therefore, this result shows that disproportionately more interactions than species are lost with the removal of harvested species. In addition, the removal of harvested species decreased the fraction of omnivore species and the mean trophic level but this decrease is not different to that produced by the random removal of the same number of species (Table S2), suggesting that the impact of artisanal fisheries may not be strong enough to destabilize^6^ and shorten^7^ the intertidal food web. Finally, the number of secondary extinctions produced by the removal of harvested species was less than the average number of secondary extinctions obtained when removing the same number of species but selected at random (Table S2). These results reinforce the observation that artisanal fisheries share prey with other consumers that also forage on other non-harvested species in the food web, so alternative resources remain when all harvested species go extinct.

### **Model Parametrization**

Supplementary Table S4 shows all the model parameters with their initial values and descriptions.

We used empirical measures of species body size, which was extracted from [2], to allometrically parameterize intrinsic growth rate of autotroph, as well as metabolic rate, and maximum consumption rate of each species (see Methods in main text).

The initial biomass was estimated from density (mobile species + cnidaria) and the surface cover (sessile species) recorded during six years of sampling in the Chilean marine reserve “Estacion Costera de Investigaciones Marinas” (ECIM) in central south of Chile. The density of mobile species was multiplied by the species average body size. For sessile species, the surface cover was multiplied by the weight of each species per unit of cover (e.g. [8]).

We calculated the community-level carrying capacity, *K,* by dividing all the primary producers into six functional groups (microalgae, ephemerals, corticates, crustoses, corallines, and kelps)^4^. In each functional group, we multiplied the biomass of the species exhibiting the highest growth rate with the number of species that compose its functional group. Finally, we summed the resulting biomass over all functional groups.

Based on empirical experiments in aquatic^9^ and terrestrial ecosystems^10^, we assumed that the half-saturation density parameter (B0) decreases with increasing trophic levels in 10^3^ order of magnitude. We use the values of Boit et al.^11^ as a reference value. For herbivores, we fit the B0 to ensure their persistence. For the other parameters, we used values from Calbet, A. & Saiz ^9^ (see Supplementary Table S4).

### **References***:*

1. Thiel, M. *et al.* The Humboldt current system of northern and central Chile: oceanographic processes, ecological interactions and socioeconomic feedback. in *Oceanography and Marine Biology* (eds. Gibson, R., Atkinson, R. & Gordon, J.) vol. 20074975 195–344 (CRC Press, 2007).

2. Kéfi, S. *et al.* Network structure beyond food webs: mapping non-trophic and trophic interactions on Chilean rocky shores. *Ecology* **96**, 291–303 (2015).

3. Kéfi, S., Miele, V., Wieters, E. A., Navarrete, S. A. & Berlow, E. L. How Structured Is the Entangled Bank? The Surprisingly Simple Organization of Multiplex Ecological Networks Leads to Increased Persistence and Resilience. *PLoS Biol* **14**, e1002527 (2016).

4. Lurgi, M. *et al.* Geographical variation of multiplex ecological networks in marine intertidal communities. *Ecology* (2020) doi:10.1002/ecy.3165.

5. Williams, R. J. & Martinez, N. D. Limits to Trophic Levels and Omnivory in Complex Food Webs: Theory and Data. *The American Naturalist* **163**, 458–468 (2004).

6. Kuparinen, A., Boit, A., Valdovinos, F. S., Lassaux, H. & Martinez, N. D. Fishing-induced life-history changes degrade and destabilize harvested ecosystems. *Sci Rep* **6**, 22245 (2016).

7. Pauly, D. Fishing Down Marine Food Webs. *Science* **279**, 860–863 (1998).

8. Wieters, E. A., Broitman, B. R. & Brancha, G. M. Benthic community structure and spatiotemporal thermal regimes in two upwelling ecosystems: Comparisons between South Africa and Chile. *Limnol. Oceanogr.* **54**, 1060–1072 (2009).

9. Calbet, A. & Saiz, E. Effects of trophic cascades in dilution grazing experiments: from artificial saturated feeding responses to positive slopes. *Journal of Plankton Research* **35**, 1183–1191 (2013).

10. Mulder, C. & Hendriks, A. J. Half-saturation constants in functional responses. *Global Ecology and Conservation* **2**, 161–169 (2014).

11. Boit, A., Martinez, N. D., Williams, R. J. & Gaedke, U. Mechanistic theory and modelling of complex food-web dynamics in Lake Constance: Mechanistic modelling of complex food web dynamics. *Ecology Letters* **15**, 594–602 (2012).

12. Gómez-Canchong, P., Quiñones, R. A. & Brose, U. Robustness of size–structure across ecological networks in pelagic systems. *Theor Ecol* **6**, 45–56 (2013).

13. Brose, U., Williams, R. J. & Martinez, N. D. Allometric scaling enhances stability in complex food webs. *Ecol Letters* **9**, 1228–1236 (2006).

14. Testa, G., Masotti, I. & Farías, L. Temporal variability in net primary production in an upwelling area off central Chile (36°S). *Front. Mar. Sci.* **5**, 179 (2018).

15. Williams, Rich J. Network 3D: visualizing and modelling food webs and other complex networks. *Microsoft Research, Cambridge, UK* (2010).

### **Tables**

**Table S1**. Rank of the 30 most connected species of the intertidal food web

| **Rank** | **Species name** | **Harvested** | **Total number of interactions (degree)** |
| --- | --- | --- | --- |
| 1 | *Fissurella limbata* | yes | 67 |
| 2 | *Fissurella crassa* | yes | 63 |
| 3 | *Acanthopleura echinata* | yes | 60 |
| 4 | *Fissurella costata* | yes | 60 |
| 5 | *Chiton granosus* | yes | 59 |
| 6 | *Chiton latus* | no | 57 |
| 7 | *Chiton cummingii* | no | 56 |
| 8 | *Enoplochiton niger* | no | 56 |
| 9 | *Chaetopleura peruviana* | no | 52 |
| 10 | *Fissurella cummingii* | yes | 47 |
| 11 | *Heliaster helianthus* | no | 43 |
| 12 | *Scurria araucana* | no | 41 |
| 13 | *Siphonaria lesoni* | no | 41 |
| 14 | Gulls | no | 41 |
| 15 | *Acanthocyclus gayi* | no | 40 |
| 16 | *Fissurella maxima* | yes | 39 |
| 17 | *Tegula atra* | yes | 39 |
| 18 | *Tonicia benaventii* | no | 39 |
| 19 | *Tonicia chilensis* | no | 39 |
| 20 | *Tonicia elegans* | no | 39 |
| 21 | *Scurria ceciliana* | no | 38 |
| 22 | *Scurria variabilis* | no | 38 |
| 23 | *Fissurella picta* | yes | 37 |
| 24 | *Fissurella puhlcra* | yes | 37 |
| 25 | *Scurria plana* | no | 37 |
| 26 | *Scurria viridula* | no | 37 |
| 27 | *Acanthocyclus hassleri* | no | 36 |
| 28 | *Balanus laevis* | no | 36 |
| 29 | *Nothobalanus flosculus* | no | 36 |
| 30 | *Lottia orbigny* | no | 35 |

**Table S2.** Structural properties of the intertidal food web before and after removing all 22 harvested species (i.e., “After

non-random removal”), and after removing the same number of species randomly (i.e., “After random removal”). The “After random removal” column shows the mean and 95% confidence interval of structural property values of 1000 iterations of randomly removing 22 species out of the 107 species in the food web. The fourth column represents the percentage of change of each structural property, calculated as [(“after non-random removal” – “before any species removal”)/ “before any species removal”]. The sixth column indicate whether the change of the structural properties produced by the loss of harvested species (non-random deletion) is different to the changes produced by random deletions. MeanSWTL is the short-weighted trophic level of a food web.

| Structural property | Before any species removal | After  non-random removal | Percentage of change (%) | After  random  removal | Non-random  vs  random removal |
| --- | --- | --- | --- | --- | --- |
| Richness | 107 | 85 | -21 | 82 ± 0.4 | ≠ |
| Number of links | 1381 | 718 | -48 | 819 ± 6 | ≠ |
| Connectance | 0.12 | 0.10 | -18 | 0.13 ± 0 | ≠ |
| %-Omnivore | 37 | 32 | -12 | 32 ± 0 | = |
| MeanSWTL | 1.64 | 1.62 | -1 | 1.62 ± 0.01 | = |

**Table S3.** F_max_ values used to produce the total biomass decrease of the harvested basal species, harvested filter-feeders, and the other harvested species in a -50%, -80% and -100% with respect of their original biomass before a fishing scenario. Due to the interspecific variation in each trophic category is very low (Fig. S3), we did not look for the value of F_max_ necessary to reduce the biomass of each of the species separately. F_max_ = 0.001 means that fisheries remove 0.1% of the available biomass of the harvested species; while F_max_ = 1 means that fisheries remove 100% of the available biomass of the harvested species.

| Type of harvested species | Biomass decrease of interest in harvested species | F_max_ value that produce the biomass decrease of interest |
| --- | --- | --- |
| Basal species |  |  |
|  | 50% | 0.00125 |
|  | 80% | 0.0022 |
|  | 100% | 0.01 |
| Filter-feeders and herbivores |  |  |
|  | 50% | 0.23 |
|  | 80% | 0.8 |
|  | 100% | 1 |
| Others consumers |  |  |
|  | 50% | 0.23 |
|  | 80% | 0.8 |
|  | 100% | 1 |

**Table S4**. Initial parameter values used in our version of the Allometric Trophic Network (ATN) model. In references column ECIM refers to the coastal marine research station of the Pontificia Universidad Catolica de Chile.

| Parameter | Unit of measurement | Definition | Initial values  min., max | References |
| --- | --- | --- | --- | --- |
| B | g / m^2^ | Population abundance | 1.26 x 10^-4^, 112107 | Empirical values from ECIM, Diatoms, and Plankton values from [12] |
| M | g | Body mass | 1 x 10^-5^, 500 | [2] |
| r | 1 / day | Mass-specific growth rate of basal species | 0.1075, 3.76 | Calculated using [13] |
| x | 1 / day | Mass-specific metabolic rate of consumers | 0.7284, 70.96 | Calculated using [13] |
| y | - | Maximum consumption rate | 1, 5.8 | Calculated using [13] |
| K | g / m^2^ | Carrying capacity of basal species | 176299 | K adapted from [11] |
| c | - | Competition coefficient of basal species | 1 | [11] |
| fa | - | The fraction of biomass that is assimilated from consumer | 0.4 | [11] |
| fm | - | The fraction of biomass that is respired to metabolic maintenance | 0.1 | [11] |
| e | - | Assimilation efficiency | 0.45, 0.85 | [13] |
| d | m^2^ / g | Intra-specific interference | 0.5 | [11] |
| q | - | Functional response | 1.2 | [11] |
| ω | - | Resources preference | 1/n_resources_ | [11] |
| p | - | Fractions of shared resources | 0, 1 | [11] |
| B0 | g / m^2^ | Half-saturation density | 150, 15000 | Adapted from [11] |
| s | g / m^2^ | The subsidy that is considered in plankton dynamic. | 12% of the initial biomass | [14] |

**Table S5**. Species name corresponding to the species number (Code column) shown in the x-axis of Supplementary Figs. S3 and S5.

| Code | Species Name | Code | Species Name | Code | Species Name |
| --- | --- | --- | --- | --- | --- |
| 1 | *Acanthina monodon* | 47 | *Scurria viridula* | 93 | *Peysonella* spp. |
| 2 | *Concholepas concholepas* | 48 | *Scurria zebrina* | 94 | *Plocamium cartilageneum* |
| 3 | *Acanthopleura echinata* | 49 | *Siphonaria lessoni* | 95 | *Prionitis* spp. |
| 4 | *Chiton granosus* | 50 | *Echinolittorina peruviana* | 96 | *Gastroclonium cyclindricum* |
| 5 | *Fissurella costata* | 51 | *Austrolittorina araucana* | 97 | *Rhodymenia* sp. |
| 6 | *Fissurella crassa* | 52 | *Onchidella* sp*.* | 98 | *Schottera nicaensis* |
| 7 | *Fissurella cummingi* | 53 | *Balanus laevis* | 99 | *Schyzimenia doryophora* |
| 8 | *Fissurella limbata* | 54 | *Jhelius cirratus* | 100 | *Trematocarpus* spp. |
| 9 | *Fissurella maxima* | 55 | *Nothobalanus flosculus* | 101 | *Corallina offcinalis v*ar. Chilensis |
| 10 | *Fissurella picta* | 56 | *Nothochthamalus scabrosus* | 102 | *Hildenbrandia lecanelieri* |
| 11 | *Fissurella puhlcra* | 57 | *Brachidontes granulata* | 103 | *Lithothamnion* spp*.* |
| 12 | *Scurria scurra* | 58 | *Perumytilus purpuratus* | 104 | *Ralfsia californica* |
| 13 | *Tegula atra* | 59 | *Semimytilus algosus* | 105 | Benthic diatoms |
| 14 | *Austromegabalanus psittacus* | 60 | *Allelopetrolisthes punctatus* | 106 | Plankton |
| 15 | *Pyura chilensis* | 61 | *Petrolisthes spinifrons* |  |  |
| 16 | *Durvillaea antarctica* | 62 | *Petrolisthes angulosus* |  |  |
| 17 | *Lessonia nigrescens* | 63 | *Petrolisthes tuberculatus* |  |  |
| 18 | *Gelidium rex* | 64 | *Petrolisthes tuberculosus* |  |  |
| 19 | *Sarcothalia* spp. | 65 | *Phragmatopoma* spp*.* |  |  |
| 20 | *Mazzaella laminarioides* | 66 | *Bryopsis* spp. |  |  |
| 21 | *Pyropia* spp. | 67 | *Centroceras* spp. |  |  |
| 22 | *Ulva rigida* | 68 | *Ceramium* spp*.* |  |  |
| 23 | Gulls | 69 | *Chaetomorpha* spp. |  |  |
| 24 | *Cinclodes nigrofumosus* | 70 | *Cladophora* spp*.* |  |  |
| 25 | *Anthotoe* spp. | 71 | *Ectocarpus silicosus* |  |  |
| 26 | *Bunodactis* spp*.* | 72 | *Enteromorpha compressa* |  |  |
| 27 | *Oulactis concinnata* | 73 | *Halopteris funicularis* |  |  |
| 28 | *Parantheopsis* spp. | 74 | *Polysiphonia* spp. |  |  |
| 29 | *Phymactis* spp. | 75 | *Rhizoclonium* ambiguum |  |  |
| 30 | *Trimusculus peruvianus* | 76 | *Scythosiphon lomentaria* |  |  |
| 31 | *Heliaster helianthus* | 77 | *Ulvella* spp. |  |  |
| 32 | *Stichaster striatus* | 78 | *Adenocystis utricularis* |  |  |
| 33 | *Acanthocyclus gayi* | 79 | *Ahnfeltiopsis* spp*.* |  |  |
| 34 | *Acanthocyclus hassleri* | 80 | *Chondrus canaliculatus* |  |  |
| 35 | *Chaetopleura peruviana* | 81 | *Codium dimorpha* |  |  |
| 36 | *Chiton cummingsi* | 82 | *Colpomenia phaeodactyla* |  |  |
| 37 | *Chiton latus* | 83 | *Colpomenia sinuosa* |  |  |
| 38 | *Enoplochiton niger* | 84 | *Gelidium* spp*.* |  |  |
| 39 | *Tonicia lineolata* | 85 | *Glossophora kunthii* |  |  |
| 40 | *Tonicia chilensis* | 86 | *Grateloupia* spp. |  |  |
| 41 | *Tonicia elegans* | 87 | *Gymnogongrus furcellatus* |  |  |
| 42 | *Lottia orbignyi* | 88 | *Laurencia chilensis* |  |  |
| 43 | *Scurria araucana* | 89 | *Montemaria horridula* |  |  |
| 44 | *Scurria ceciliana* | 90 | *Nothogenia* spp. |  |  |
| 45 | *Scurria plana* | 91 | *Petalonia Fascia* |  |  |
| 46 | *Scurria variabilis* | 92 | *Petroglossum* spp*.* |  |  |

**
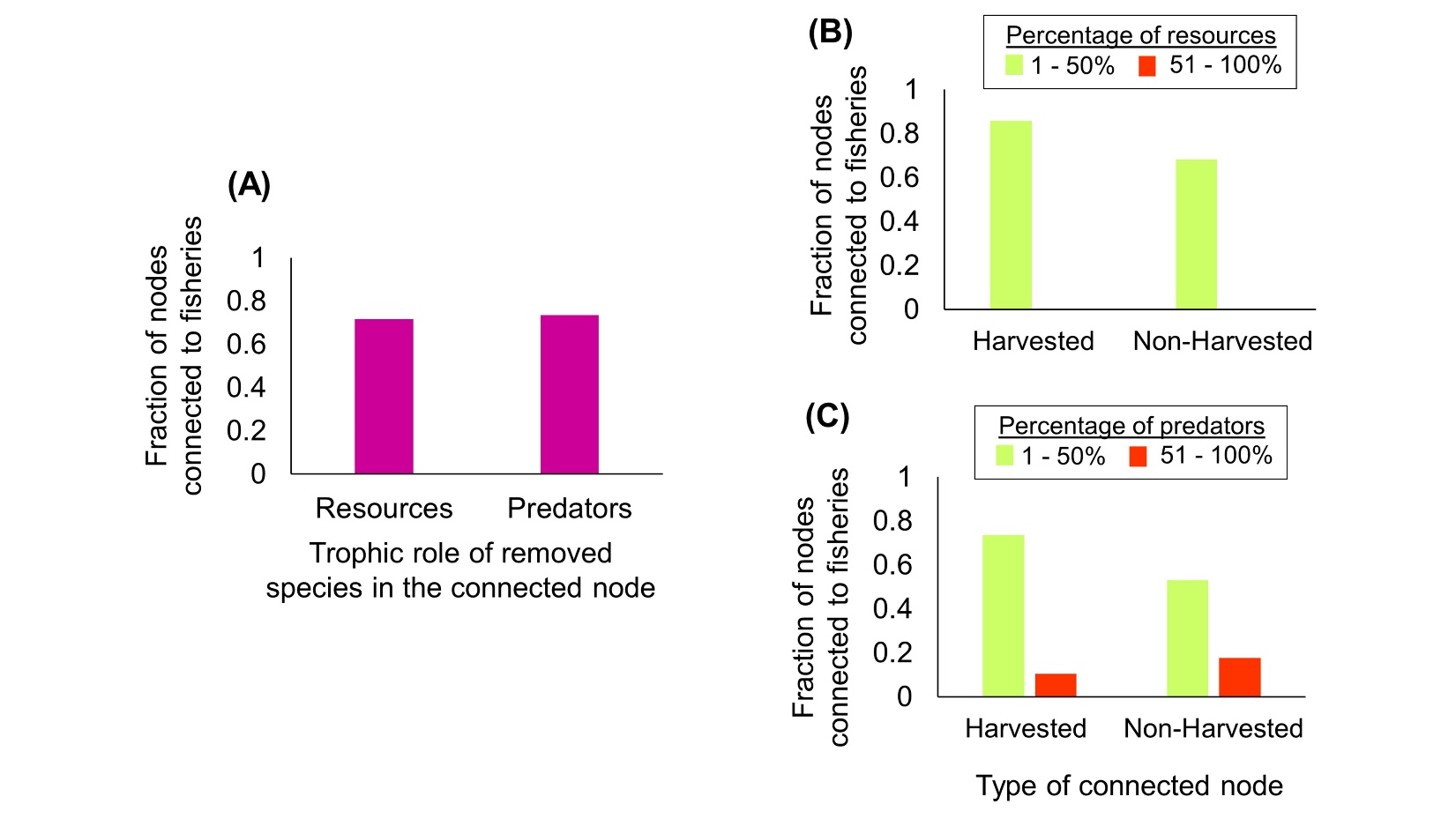
Fig. S1**.

**Figure S1**. (A) Fraction of species in the food web that are trophically connected (at least once) with harvested species that are either a resource and/or a predator (x-axis). Each purple bar presented in (A) is further divided in: (B) the percentage of resources shared with fisheries by the harvested and non-harvested consumers of the food web, and (C) the percentage of predators of harvested and non-harvested species extracted by fisheries. In B and C, the bars represent the breadth (1-50% in grenn and 51-100% in red) of the resource species (B) and predator species (C) of harvested and non-harvested species that are harvested by artisanal fisheries on.

**Fig. S2**.





**Figure S2**. Food web robustness (R_50_) to three deletion sequences and using a static (yellow circle) vs. dynamical (grey circle) approach. The performed deletion sequences removed species: (1) randomly (hereafter “random”), (2) from the most to the least connected species (hereafter “most-connected”), and (3) from the most connected species that trophically support highly connected species to the least connected species supporting low connected species (hereafter “Supporting-basal”). In the case of the random deletion sequence, the circles represent the average and the error bars the ± 95. C.I. of 1000 random deletion sequences.

**Fig. S3**


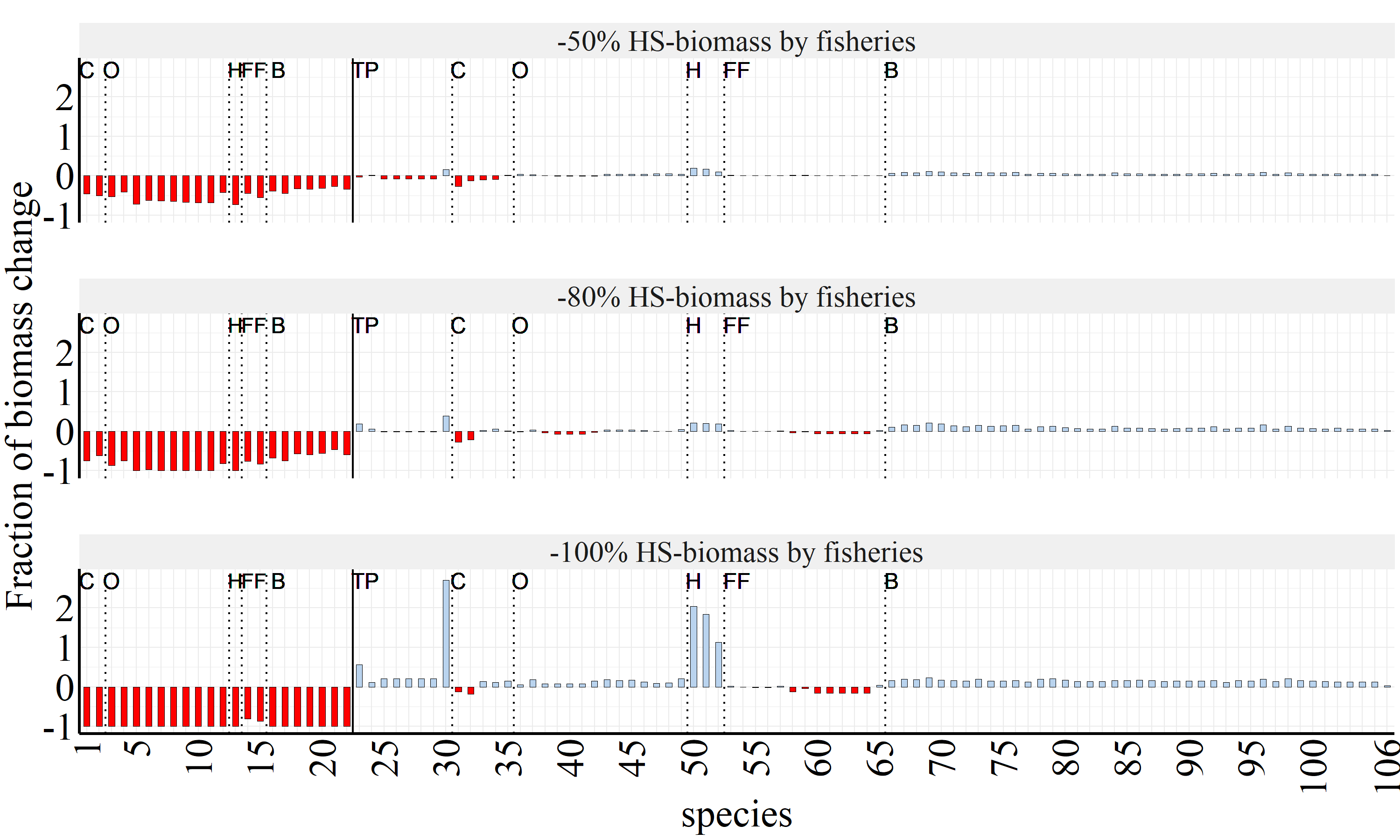


**(A)**

**(B)**

**(C)**

**Figure S3.** Fraction of biomass change (y-axis) of each species (x-axis) of the intertidal food web after simultaneously reducing the biomass of all harvested species in a -50% (A), -80% (B) and -100% with respect to their original biomass before a fishing scenario (C). Red bars represent negative effects on species biomass, while blue bars represent positive effects. From the bold vertical line to the left, the figure shows all the harvested species (HS). From the bold line to the right, the figure shows all the non-harvested species. Species are organized by trophic level (TP: top-predators, C: carnivores, O: omnivores, H: herbivores, FF: filter-feeders, B: basal species) and their identity can be found by matching their number id with the numbers in Supplementary Table S5.

**Fig. S4**


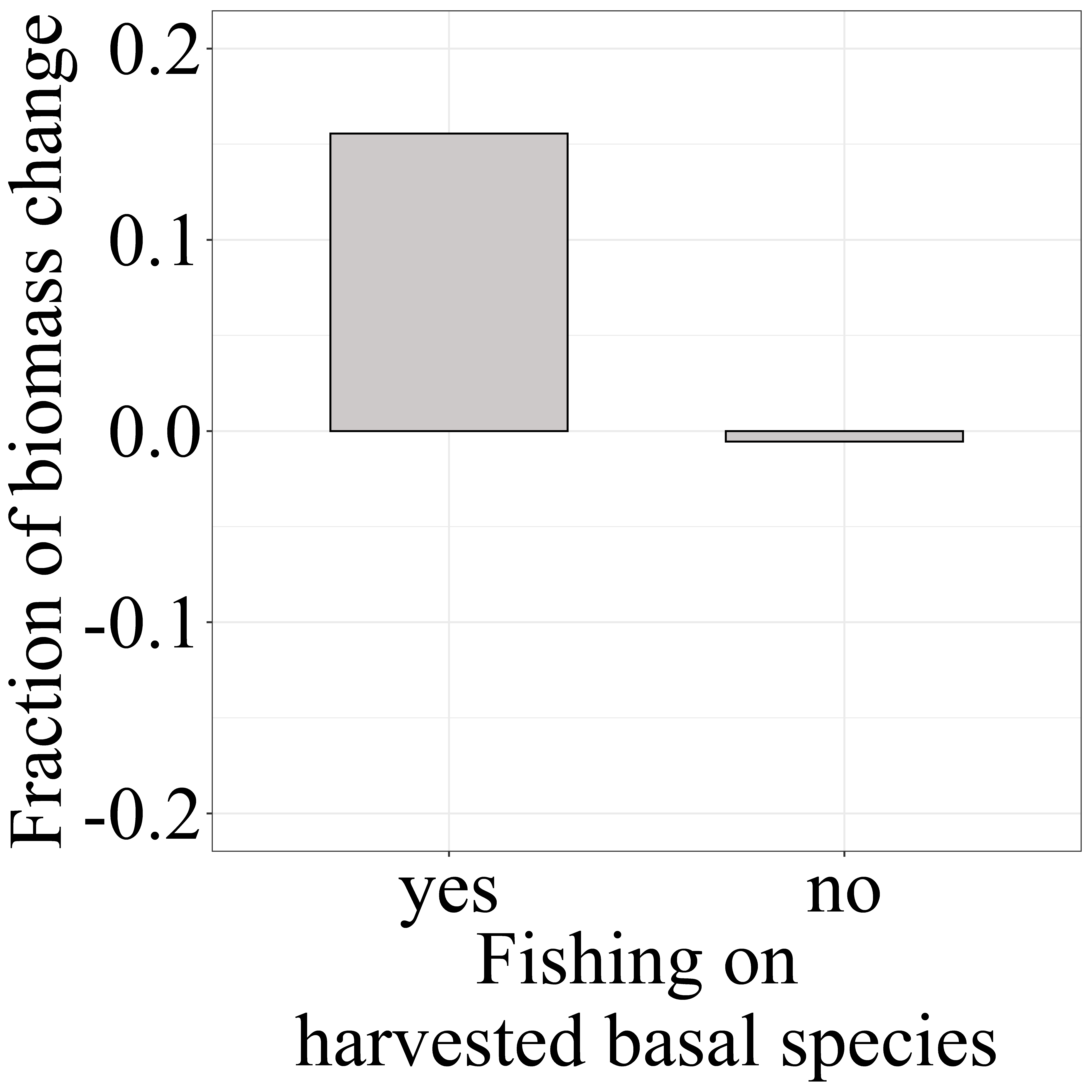


**Figure S4.** Contribution of harvested basal species over the biomass of non-harvested basal species. The figure represents the fraction of the total biomass change of non-harvested basal species (y-axis) after fishing with the maximum exploitation rate (F_max_ = 1) for all harvested species, including (yes) and without including (no) harvested macroalgae in the list of harvested species.

**Fig. S5**


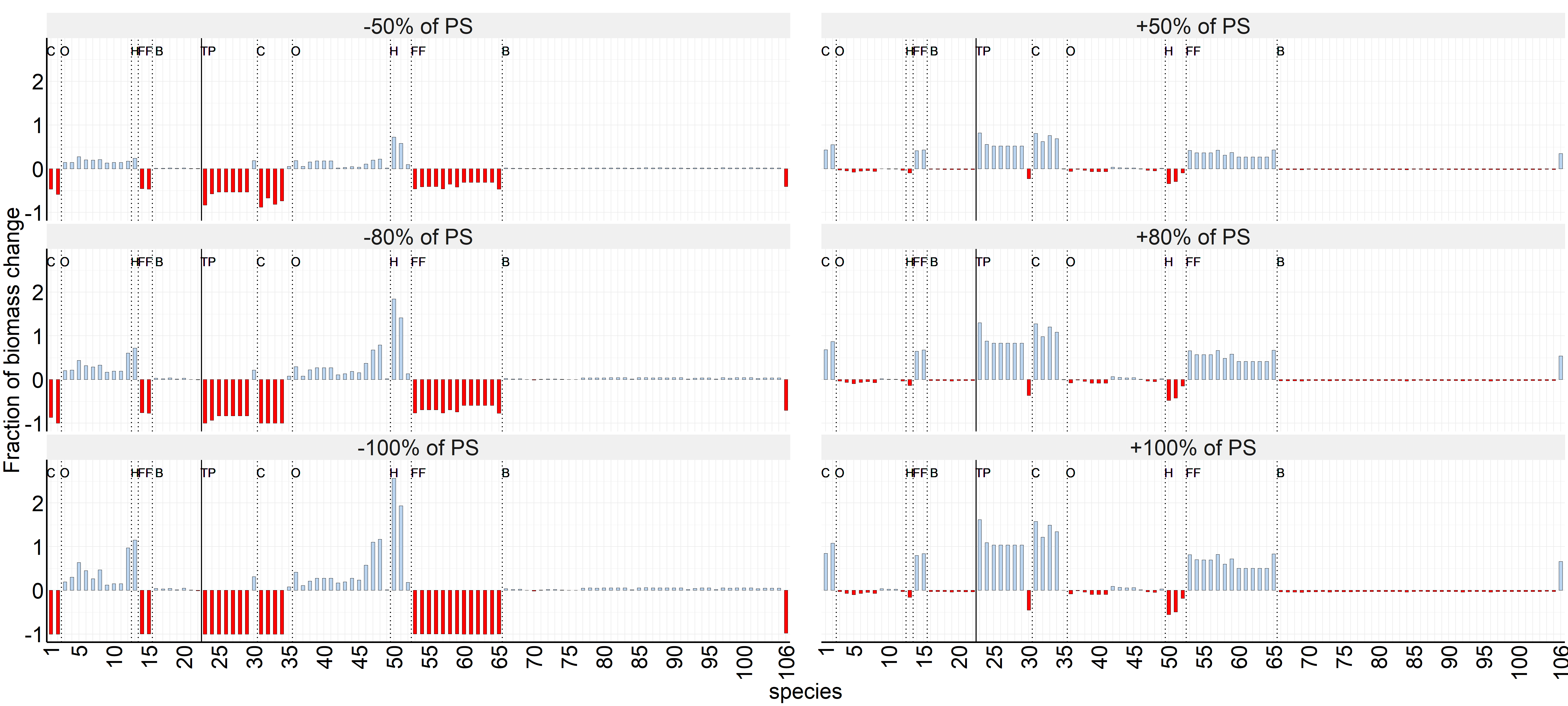


**(A)**

**(B)**

**(C)**

**(D)**

**(E)**

**(F)**

**Figure S5.** Fraction of biomass change (y-axis) of each species (x-axis) of the intertidal food web after perturbing plankton subsidy (species 106) in -100% (E), -80% (C), -50% (A), +50% (B), +80% (D), +100% (F) of their basal productivity. The red bar represents negative effects on species biomass, while blue bars represent positive effects. From the bold vertical line to the left, the figure shows all the harvested species. From the bold line to the right, the figure shows all the non-harvested species. Species are organized by trophic level and their identity can be found by matching their number id with the numbers in Supplementary Table S5.

**Fig. S6**

**
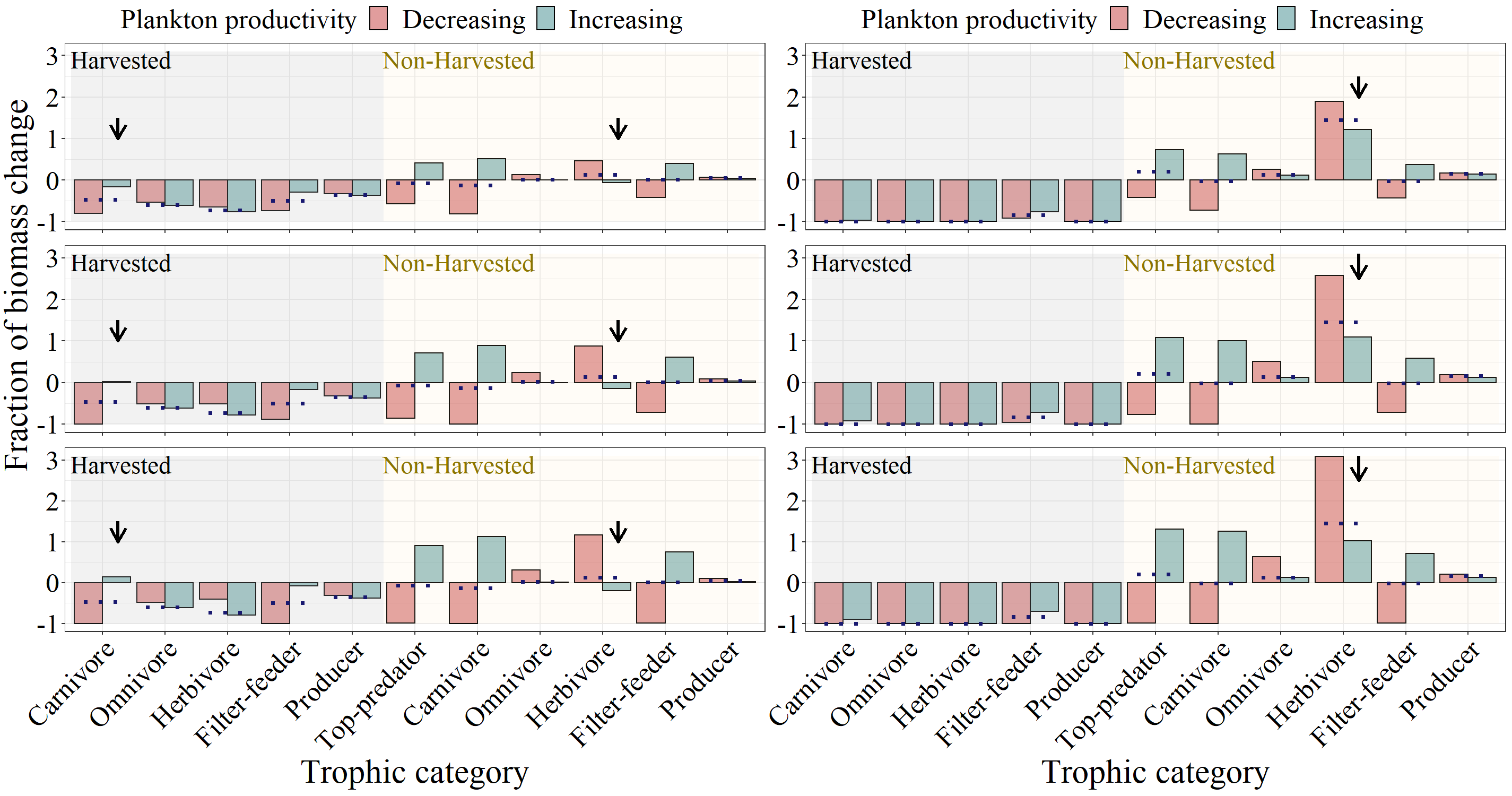
**

50% of PP

100% of PP

80% of PP

**(E)**

**(F)**

**(C)**

**(A)**

- 100% HS-biomass by fisheries

- 50% HS-biomass by fisheries

**(D)**

**(B)**

**Figure S6.** Combined effects of artisanal fisheries and plankton-productivity alterations on food web dynamics. Fraction of total biomass change (y-axis) of each trophic category (x-axis) after decreasing (red bars) and increasing (blue bars) the plankton productivity (PP) in ±50% (A and B), ±80% (C and D), and ±100% (E and F), and after decreasing the biomass of all harvested species (HS) in a -50% (A and C) and in a -100% (B and D). The grey and yellow shading represent the biomass change of harvested and non-harvested species, respectively. The arrows highlight the most remarkable changes between the two levels of plankton subsidy perturbation and the two levels of fishing. The dotted lines represent the independent effect fishing (i.e., without plankton subsidy perturbation) on the biomass of each trophic category as a reference point. The results for the -80% fishery model are not in the figure since the direction of the changes are similar with -100% fishing scenario, they only change in magnitude (see Fig. 2B and C).

**Fig. S****7**

**Figure S7.** Shallow-subtidal marine Chilean food web. (A) Color node represents the harvested (red) and non-harvested (yellow) species. Letter F represents the fisheries node. Node size represents the number of trophic interactions (degree) of each node. Bottom nodes represent basal species, and the nodes of the top represent top predators. The left axes represent the trophic level (obtained from the minimum to the maximum value of the SWTL used to calculate the MeanSWTL) from the minimum to the maximum trophic level. Drawn using Network3D software^15^. Harvested species was recognized from the official governmental webpage of the Chilean fishing service ([www.sernapesca.cl](http://www.sernapesca.cl)). (B) Fraction of secondary extinctions (y-axis) produced in this food web after the sequential removal of species (x-axis) with a static approach. Gray and red circle represent most-connected and supporting-basal deletion sequence, while blue tringle represents harvesting deletion sequence. In the random deletion sequence, circles represent the average and the error bars represent the 95% confidence interval of 1000 simulations.
